# Selective detection of arsenite in alkaline media using a yolk-shell (Ce/Fe) terephthalate/organoclay framework stripping voltammetric sensor

**DOI:** 10.1007/s00604-025-07782-x

**Published:** 2026-03-17

**Authors:** Mona Elfiky, Amr M. Beltagi

**Affiliations:** 1 Department of Chemistry, Faculty of Science, Tanta University, Tanta, 31527 Egypt; 2 Department of Chemistry, Faculty of Science, Kafrelsheikh University, Kafrelsheikh, 33516 Egypt

**Keywords:** Cerium, Ferric, Montmorillonite, Arsenite ions, Square wave adsorptive stripping voltammetry, Modified graphite paste sensor

## Abstract

To address environmental and health challenges while promoting sustainable clean water and soil, a highly sensitive graphite paste sensor incorporating a nanocomposite material has been developed. This sensor utilizes a yolk-shell (Ce/Fe) terephthalate (Ce/Fe-Tph.) framework intercalated with organoclay (MMt_D2000_) nanolayers, forming a Ce/Fe-Tph./MMt_D2000_ framework nanocomposite for the detection of arsenite (As^3+^) ions. The yolk-shell Ce/Fe-Tph./MMt_D2000_ framework nanocomposite was synthesized via a solvothermal method, yielding excellent crystallinity and a high surface area of 1054 m².g^− ^¹. This enhanced surface area provides abundant active sites, thereby improving sensor performance and supporting sustainable environmental monitoring. The 1.0% (Ce/Fe-Tph./MMt_D2000_) modified graphite paste sensor (MGPS) exhibited enhanced electrocatalytic activity and adsorption performance. It achieved a broad detection range of 0.0015–0.0749 ppb (0.02–1.0 nM) and an ultralow detection limit of 0.00045 ppb (0.006 nM) for As^3+^ ions using square wave adsorptive cathodic stripping voltammetry (SW-AdCSV), with a sensitivity of 436.68 µA/ppb (32.7 µA.nM^− 1^). The sensor also demonstrated high accuracy, reproducibility, and stability, along with exceptional selectivity for the detection of As^3+^ ions in the presence of other heavy metal ions, without interference in strongly basic media. This sensor provides a simple, direct method for detecting As^3+^ ions in environmental samples including real water and soil matrices, without requiring sample pretreatment. Its capabilities align with sustainable development goals by advancing environmental monitoring.

**Graphical Abstract:**

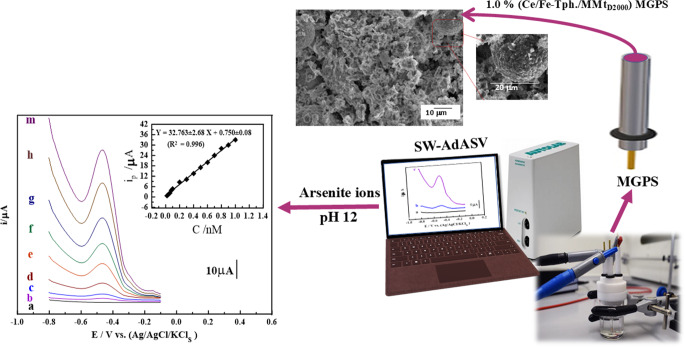

**Supplementary information:**

The online version contains supplementary material available at 10.1007/s00604-025-07782-x.

## Introduction

Heavy metal ions (HMIs) are harmful to the ecosystem when their concentrations exceed the prescribed limits. They possess carcinogenic properties and can lead to significant health complications. Thus, the rapid and precise detection of metal ions is essential. The metal ions arsenic (As^3+^), cadmium (Cd^2+^), chromium (Cr^3+^), lead (Pb^2+^), and mercury (Hg^2+^) are considered among the most toxic and carcinogenic HMIs [1, 2]. These metals may remain in the environment for decades or even more because they are not biodegradable. Once heavy metals enter the human body through ingestion, inhalation, or skin contact, they can induce adverse effects, including nausea, vomiting, diarrhea, and harmful effects even at low-level or short-term exposure [3].

Arsenic is one of the most hazardous heavy metals for both humans and animals, posing significant environmental risks and threatening the lives of millions [4]. Among its various chemical forms arsenite, arsenate, and organic derivatives, its inorganic states are more toxic due to their higher ionic mobility compared to organic species. Moreover, As^III^ (arsenite) is generally more toxic than As^V^ (arsenate). The World Health Organization recommends reducing arsenic concentrations in natural and drinking water to below 10 ppb (0.14 µM) for safety [5–7]. Traditional arsenic detection methods in water [8], including inductively coupled plasma-mass spectrometry [9, 10], atomic absorption and emission spectroscopy [11], non-atomic spectrometry [12], atomic fluorescence spectroscopy [13], integrated colorimetry [14], and high-performance liquid chromatography [15], require expensive equipment, skilled personnel, and complex sample preparation. Additionally, these methods involve time-consuming analyses, making them impractical for field applications. In contrast, electrochemical sensors have gained attention in recent years as promising alternatives for both qualitative and quantitative detection of heavy metals [16–18]. These methods offer advantages such as simplicity, high sensitivity, robustness, selectivity, and ease of use. Furthermore, electrochemical systems can be miniaturized, making them suitable for on-site use in various environments. The characteristics of the sensor surface significantly impact the electrochemical process, and modifying the sensor surface with appropriate reagents can enhance sensitivity and selectivity. Recent research has focused on developing electrochemical sensors for arsenic detection, leading to numerous methods for modifying sensors to improve performance [19–23]. Studies confirm electrochemical sensors are effective for detecting As^3+^ ions by measuring electrical changes. Modified sensors such as those using gold or thoria nanoparticles, silica composites, or graphene-based materials have achieved low detection limits between 0.025 ppb and 0.93 µg/L [24–29]. However, many of these methods suffer from interference by other metal ions, and show limited linear detection ranges. Additionally, their use in real environmental samples is not yet fully validated. These challenges emphasize the need for new approaches that combine high sensitivity, low detection limits, and strong resistance to interference.

Metal–organic frameworks (MOFs) are emerging as promising materials for the development of adsorbents with high adsorption capacity and well-defined porosity. These frameworks are synthesized by linking various metal ions with flexible organic linkers through a coordination process, enabling the creation of materials with desirable properties such as high specific surface area, tunable pore size, and structural stability [30–37]. Moreover, MOFs derived from multi-component organic ligands exhibit significant potential for a wide range of applications. In particular, cerium-based (Ce-based) MOFs have gained attention in electrochemical and photocatalytic applications due to their large surface area, variable valence states (Ce⁴⁺ to Ce³⁺), excellent redox properties, adsorption capabilities, and catalytic performance [38, 39]. For instance, Dong et al. developed a Ce-MOF biosensor for the detection of telomerase, while Chen et al. created an electrochemical sensor using a Ce-MOF electrocatalyst to enhance the detection of thrombin. Recently, Elfiky et al. developed a modified stripping voltammetric sensor based on Ce-BDC MOF nanoparticles for the detection of curcumin (CUR), achieving a low detection limit of 6 pM.

Moreover, MIL-101(Fe) is a porous material with a cage-like structure formed by the self-assembly of iron ions and terephthalic acid molecules [40]. This unique structure imparts excellent hydrophilicity and chemical stability, making it suitable for use in biosensors targeting various species [41]. Additionally, it demonstrates strong interactions, such as π-π stacking, hydrogen bonding, and electrostatic forces, with negatively charged nucleic acid sequences, which enhances its ability to capture pollutants and significantly improves the sensitivity of electrochemical sensors [42]. However, the low electrical conductivity of MOF may limit their electrochemical applications. Therefore, the development of heterometallic centers in bimetallic MOFs enables synergistic effects through the integration of a second metal ion, enhancing electrochemical properties through lattice distortions, electronic coupling, and improved porosity [43]. The strong coordination interaction between Fe-MOF and arsenic pollutants renders Fe-MOF highly adsorptive toward As^3+^ ions [44, 45], despite its poor electronic conductivity, which may limit its performance. To enhance conductivity, MOF-based composites are commonly designed by adding conductive materials like carbon substrates [45], polymers [46], and MXene [47]. However, to date, there have been no comprehensive reports on the use of a modified stripping voltammetric sensor based on a co-mixed Ce/Fe-Tph. framework for the electrochemical detection of As^3+^ ions in various fluids, including water and soil samples.

Montmorillonite (MMt), layered hydrous alumina-silicate clay, is the most common smectite clay mineral. It has an irregular lamellar structure with a diameter of 100 nm and a thickness of approximately 1 nm, composed of two layers of tetrahedral silica fused with an octahedral aluminum hydroxide layer. Exchangeable cations, such as Na^+^ in sodium-MMt, and water molecules are present in the gaps between MMt layers. These properties enable the modification of the clay by balancing the negative charge on the lamellae through isomorphic substitution. MMt stacks can incorporate guest species like metal oxides [48], metal-organic frameworks (MOFs) [37, 49], and conducting polymers to form hybrids or nanocomposites with improved physical and chemical properties for various applications. Despite these developments, there is currently no detailed report on the fabrication of nanocomposites based on a yolk-shell Ce/Fe-Tph. framework with exfoliated organoclay nanosheets (Ce/Fe-Tph./MMt_D2000_) for electrochemical sensing.

Graphite paste sensors (GPSs) offer significant benefits for miniaturized electrochemical sensors due to their cost-effectiveness, ease of preparation and modification, extensive surface area, stability, low background currents, and broad potential windows, thereby facilitating miniaturization while ensuring high sensitivity and selectivity in the detection of diverse substances [50]. Their ability to incorporate diverse modifying components facilitates the development of highly sensitive and selective sensors [50].

In this context, this work focuses on the development of a novel Ce/Fe-Tph./MMt_D2000_ framework, integrating yolk-shell Ce/Fe-Tph. with exfoliated MMt_D2000_ nanosheets using a single-step solvothermal method. A sensitive modified stripping voltammetric sensor, based on the graphite paste sensor, was developed using a yolk-shell framework as an sensor modifier. The developed sensor was applied to selectively detect arsenite ions in various environmental samples, including water and soil. The sensor demonstrated no interference from other sample components during analysis, making it highly suitable for environmental monitoring.

## Experimental part

### Materials and reagents

The reagents were used without further purification. Na-Montmorillonite (Na-MMt), sourced from Southern Clay Products (Colloid BP), Inc. (Gonzales, Texas, USA), has a cation exchange capacity of 114.8 meq/100 g. The MMt clay was dried in a vacuum oven at 100 °C for 24 h, resulting in an interlayer spacing (d_001_) of 9.6 Å. Polyoxypropylene diamine with an average molecular weight of 2000 (Jeffamine D_2000_) with a primary amine content (PAC) of 0.97 meq/g was obtained from Huntsman Corporation (TX, USA). Cerium(III) nitrate hexahydrate (Ce(NO_3_)_2_·6H_2_O, **≥** 99%), Iron(III) chloride hexahydrate (FeCl_3_·6H_2_O, **≥** 97%), terephthalic acid (Tph., **≥** 98%), potassium hexacyanoferrate(III) (K_3_[Fe(CN)_6_], **≥** 99.0%), ethanol (EtOH, **≥** 98%), ethylene glycol (EG, **≥** 99%), N, N-dimethylformamide (DMF, **≥** 98%), phosphoric acid (H_3_PO_4_, **≥** 99.0%), sodium chloride (NaCl,**≥**99.0%), sodium phosphate monobasic (NaH_2_PO_4_, **≥** 99.0%), disodium hydrogen phosphate dihydrate (Na_2_HPO_4_·2H_2_O, **≥** 99.5%), sodium phosphate (Na_3_PO_4_, **≥** 96.0%), sodium hydroxide (NaOH pellets, **≥** 98.0%), and potassium chloride (KCl, **≥** 99.0%).

Britton–Robinson universal buffer (BRB) solutions covering pH values from 2 to 12 were prepared by mixing 0.04 M solutions of boric, phosphoric, and acetic acids with 0.2 M sodium hydroxide (NaOH) in appropriate ratios. Phosphate buffer saline solutions (PBS) covering pH values from 2 to 12 were prepared by combining 50 mL of 0.1 M K_2_HPO_4_ with 50 mL of 0.1 M KH_2_PO_4_, followed by pH adjustment using 1 M HCl or 1 M NaOH. These buffers were used as supporting electrolytes. Moreover, standard solutions of As^III^, Cu^2+^, Cd^2+^, Co^2+^, Zn^2+^, Ni^2+^, Mn^2+^, Hg^2+^, Pb^2+^, Al^3+^, Cr^3+^ and Fe^3+^, were prepared by precisely diluting their stock solutions (1,000 mg/L in aqueous 0.1 M HCl, obtained from Cica, Japan). De-ionized water was obtained from a Purite-Still Plus Deionizer, connected to a Hamilton-Aqua Matic bi-distillation water system (Hamilton Laboratory Glass LTD, Kent, UK). A series of initial pH values were prepared as follows. First, 20 mL of 0.01 M NaNO_3_ solutions were adjusted to pH values ranging from 1.5 to 10 by adding 0.1 M HNO_3_ or NaOH under a nitrogen atmosphere. Then, 0.06 g of Ce/Fe-Tph./MMt_D2000_ framework nanocomposite was added to each 20 mL NaNO_3_ solution and shaken continuously for 48 h. The adsorbent was then separated by filtration, and the pH of the filtrate was recorded as pH_f_. The pH_ZPC_ of Ce/Fe-Tph./MMt_D2000_ framework nanocomposite was determined by plotting δ_pH_ (pH_f_ − pH_i_) against pH_i_.

### Characterization techniques

To investigate the surface morphology of the synthesized nanocomposites, field emission scanning electron microscopy (FE-SEM, Quanta™ 250) was employed. The HR-TEM analysis utilized a carbon-coated copper grid (200 mesh) for sample support. Fourier transform infrared (FT-IR) spectra were recorded using both a PerkinElmer spectrophotometer and a Rigaku Ultima IV R185 instrument to identify functional groups and confirm chemical bonding. Crystallographic properties were assessed via X-ray diffraction (XRD) using a Rigaku Ultima IV R185 diffractometer operating with Cu-Kα radiation (λ = 1.54 Å), at 40 kV and 20 mA. Prior to surface area analysis, the nanocomposites were degassed under vacuum at 150 °C for 2 h. Specific surface area and pore size distribution were then determined using the Brunauer–Emmett–Teller (BET) method. Electrochemical properties were evaluated through impedance spectroscopy and stripping voltammetry. These measurements were carried out using a Solarton potentiostat (Model SI-1287) integrated with a frequency response analyzer (Model 1252 A, Solarton), as well as a computer-controlled potentiostat (PAR Model 263 A) for voltammetric analysis. Moreover, ZSim 3.20 software (EChem Software, Michigan, USA) was used to model analog circuit and match EIS measurements.

### Synthesis of organoclay

The organoclay **(MMt**_**D2000**_**)** was synthesized through a cation-exchange method. In brief, 1 g of Na-MMt was sonicated in 60 mL of double deionized water (DDW) and stirred for 3 h at 60 °C, followed by 24 h of stirring at 25 °C. Then, 1.24 g of acidified Jeffamine D_2000_, prepared with an aqueous HCl solution, was added to the dispersed Na-MMt solution and stirred for 24 h. The resulting product was washed thoroughly with DDW until no chloride ions (Cl⁻) were detected. Finally, the MMt_D2000_ precipitation was dried at 60 °C for 24 h.

### Synthesis of Yolk-shell Ce/Fe-Tph. framework and Ce/Fe-Tph./MMt_D2000_ framework nanocomposite

A yolk-shell Ce/Fe-Tph. Framework was synthesized using a solvothermal method as follows: 0.5 g of Ce(NO_3_)_2_·6H_2_O and 0.8 g of FeCl_3_·6H_2_O were dissolved in 45 mL of DMF and 25 mL of EG (beaker A) with constant stirring overnight. Meanwhile 0.3 g of Tph. acid was dissolved in 35 mL of DMF and 25 mL of EG (beaker B) [51]. Subsequently, the solution from beaker A was slowly added dropwise to beaker B under constant stirring for 30 min. The resulting mixture was allowed to react in an autoclave at 120 °C for 6 h. The obtained precipitate was washed thoroughly and dried in an oven at 80 °C for 12 h. A Ce/Fe-Tph./MMt_D2000_ framework nanocomposite was synthesized using the same procedure, the addition of 0.4 g of prepared organoclay (MMt_D2000_) in (beaker A), yielding 1.1 g of product.

### Fabrication of bare and modified stripping voltammetric sensors

To prepare the bare graphite paste sensor (BGPS), 5.0 g of fine graphite powder (Aldrich, 1–2 μm) was thoroughly hand mixed with 1.8 mL of paraffin oil (Sigma, d = 0.85 g/mL) in an agate mortar. The resulting homogenous paste was carefully packed into the sensor cavity (inner diameter: 3.0 mm). The surface of the BGPS was then smoothed and polished manually using clean paper until a shiny finish was achieved. The BGPS was subsequently immersed in the electrolysis cell containing an appropriate supporting electrolyte. After each voltammogram was recorded, the sensor was regenerated by altering its surface and polishing it again. For the fabrication of the Ce/Fe-Tph./MMt_D2000_ framework nanocomposite modified graphite paste sensor (MGPS) with 1.0% modifier, 4.95 g of fine graphite powder was mixed with 5.0 mg of the prepared modifier powder and 1.8 mL of paraffin oil to form a uniform paste. This process was repeated for the preparation of 1.0% Ce/Fe-Tph. The same procedure was performed utilizing 8.0 mg (1.0%), and 12.0 mg (2.0%) of the Ce/Fe-Tph./MMt_D2000_ framework nanocomposite.

### Evaluation of point of zero charge (pH_ZPC_) of Ce/Fe-Tph./MMt_D2000_

The pH_ZPC_ of Ce/Fe-Tph./MMt_D2000_ was determined using the pH drift method. Initially, 20 mL of 0.01 M NaNO₃ solutions were prepared and their pH adjusted to values ranging from 1.5 to 10 by adding 0.1 M HNO₃ or NaOH under a nitrogen atmosphere. Subsequently, 0.06 g of Ce/Fe-Tph./MMt_D2000_ was added to each solution, and the mixtures were shaken for 48 h. Then, the suspensions were filtered, and the final pH (pHₓ) of the filtrates was measured. The pH_ZPC_ was identified from the plot of ΔpH (pHₓ − pH_i_) versus pH_i_, where pH_i_ is the initial pH.

### Optimal analytical procedures

Square wave Adsorptive cathodic stripping voltammetric (SW–AdCSV) measurements were conducted using both bare and modified sensors in a standard 10 mL electrolysis cell. The cell contained a 1.0 nM solution of As³⁺ ions and a BRB or PBS as the supporting electrolyte, under optimized preconcentration conditions. Voltammograms were recorded after applying the selected accumulation time and a 5 s resting period, within the potential range (+ 0.3 to −1.1 V). Each measurement was repeated five times using fresh supporting electrolyte to ensure sensor surface renewal.

### Analysis of real water and soil samples

Brackish and coastal seawater samples were collected in polyethylene containers for analysis using the developed Ce/Fe-Tph./MMt_D2000_ MGPS. The brackish water samples were obtained from Burullus Lagoon, while the coastal seawater samples were sourced from Baltim city, Kafrelsheikh Governorate [52]. Water samples were collected a few meters from the shore and filtered through a 0.45 μm membrane filter to remove insoluble particles. The filtered samples were then diluted with double-distilled water and analyzed promptly after collection to ensure accuracy.

Three soil samples were collected at agricultural sites, located near Tanta City, Elgharbia Governorate. A 0.5 g portion of each soil sample was spiked with varying concentrations of As³⁺ ions. This mixture was then placed into a 10 mL micro-electrochemical cell containing 5.0 mL of deionized water (DDW) and 5.0 mL of buffer solution (pH 12). The system underwent sonication for 30 min to facilitate the extraction and homogenization of the analyte. Following sonication, the prepared solution was utilized for subsequent electrochemical analyses.

## Results and discussion

### Characterization of prepared modifiers

**Fig. 1:**
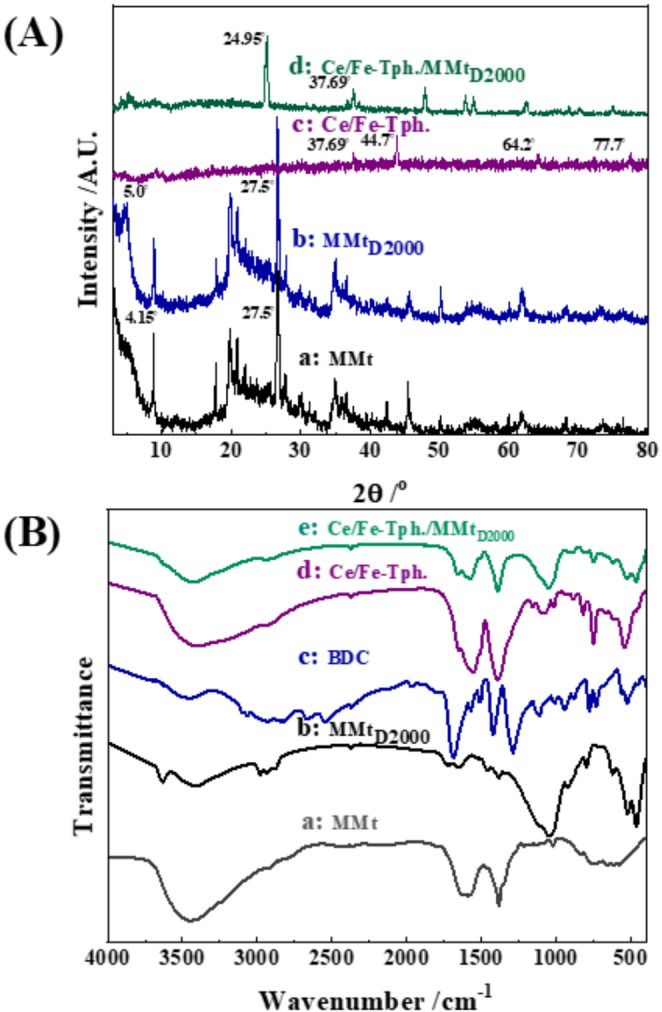
**A** XRD patterns (a) MMt, (b) MMt_D2000_, (c) Ce/Fe-Tph., and (d) Ce/Fe-Tph./MMt_D2000_ Framework. **B** FTIR spectra of (a) MMt, (b) MMt_D2000_, (c) BDC, (d) Ce/Fe-Tph., and (e) Ce/Fe-Tph./MMt_D2000_ Framework

The X-ray diffraction (XRD) patterns of MMt, MMt_D2000_, Ce/Fe-Tph., and Ce/Fe-Tph./MMt_D2000_ are displayed in [Fig. 1A] for structural analysis. In the XRD pattern of MMt, and MMt_D2000_ [Figs. 1A_a, b_], the d-spacing (d_001_) of the layers increases from 2θ ~ 5.0° (8.83 Å) for the pristine Na-MMt to 2θ ~ 4.15° (10.60 Å) for MMt_D2000_. This shift is attributed to the successful intercalation of Jeffamine D_2000_ between the layers of Na-MMt [53]. In contrast, the XRD pattern of yolk-shell Ce/Fe-Tph. framework [Fig. 1A_c_] displayed peaks at 37.54°, 44.7*°*, 64.2**°** and 77.4°, which could be related to 110, 200, 211, and 220 planes of zero-valence iron (Fe^o^) with [JCPDF No: 87–0722] [54–57]. This result suggests the formation of a zero-valent iron (Fe⁰)shell upon the surface of formed Ce/Fe-Tph. framework. On the other hand, the XRD pattern of yolk-shell Ce/Fe-Tph./MMt_D2000_ [Fig. 1A_d_] suggests that the MOF has successfully penetrated the interlayer spaces of the organoclay, resulting in changes to the basal spacing and overall diffraction profile. This implies a significant rearrangement of the MMt layers. Additionally, sharp peaks observed at 2θ ~ 24.95° and 37.69°, corresponding to Fe⁰, suggest enhanced reduction of iron species during synthesis or intercalation [57].

Furthermore, the Fourier transform infrared (FT-IR) spectra of MMt, MMt_D2000_, BDC, Ce/Fe-Tph., and Ce/Fe-Tph./MMt_D2000_ are presented in [Fig. 1B] for structural analysis. The spectrum of MMt [Fig. 1B_a_] displayed absorption bands at 3457, and 1020 cm^− 1^, which are assigned to the stretching vibration of Al-OH, and Si-O-Si, respectively [58, 59]. Additionally, bands at 1606.6, 848, and 587 cm^− 1^ are ascribed to bending of Al-OH, Al-Al-O and Si-O-Al [60], respectively [61]. The FT-IR spectrum of MMt_D2000_ [Fig. 1B_b_] displayed band shift at 1648, and 1733 cm^− 1^, which corrosponds to the asymmetrical and symmetrical bending of N-H in NH_3_^+^; thereby confirming the electrostatic attraction between silicate sheets and polyoxypropylene [62, 63]. The FT-IR spectrum of BDC [Fig. 1B_c_] exhibits characteristic bands around 1690, 1425, and 733 cm^− 1^, attributed to the stretching *v*_C=O_, *v*_C–O_, and in-plane bending *v*_Ar–H_ [64], respectively. In contrast, the FT-IR spectrum of Ce/Fe-Tph. [Fig. 1B_d_] displays a well-defined characteristic band at approximately 3448 cm^− 1^, corresponding to the stretching *v*_H–O–H,_ and *v*_OH_. As displayed in [Figs. 1B_c, d_], bands were shifted from 1690 to 1554 cm^− 1^, and the ν_COO_ stretching band shifted from 1425 to 1384 cm^− 1^. Notably, the characteristic band in-plane bending *v*_Ar–H_ band of BDC was shifted from 726 to 748 cm^− 1^, indicating the successful formation of pure Ce/Fe-Tph [37, 65]. In the FT-IR spectrum of yolk-shell Ce/Fe-Tph./MMt_D2000_ [Fig. 1B_e_], a shift in the characteristic bands of ν_COO_ stretching and in-plane bending *v*_Ar–H_ bands of BDC from 1690 to 1590 cm^− 1^, 1425 to 1392 cm^− 1^, and 726 to 752 cm^− 1^, respectively, suggest successful bonding between Ce/Fe-Tph. and exfoliated MMt clay.

**Fig. 2:**
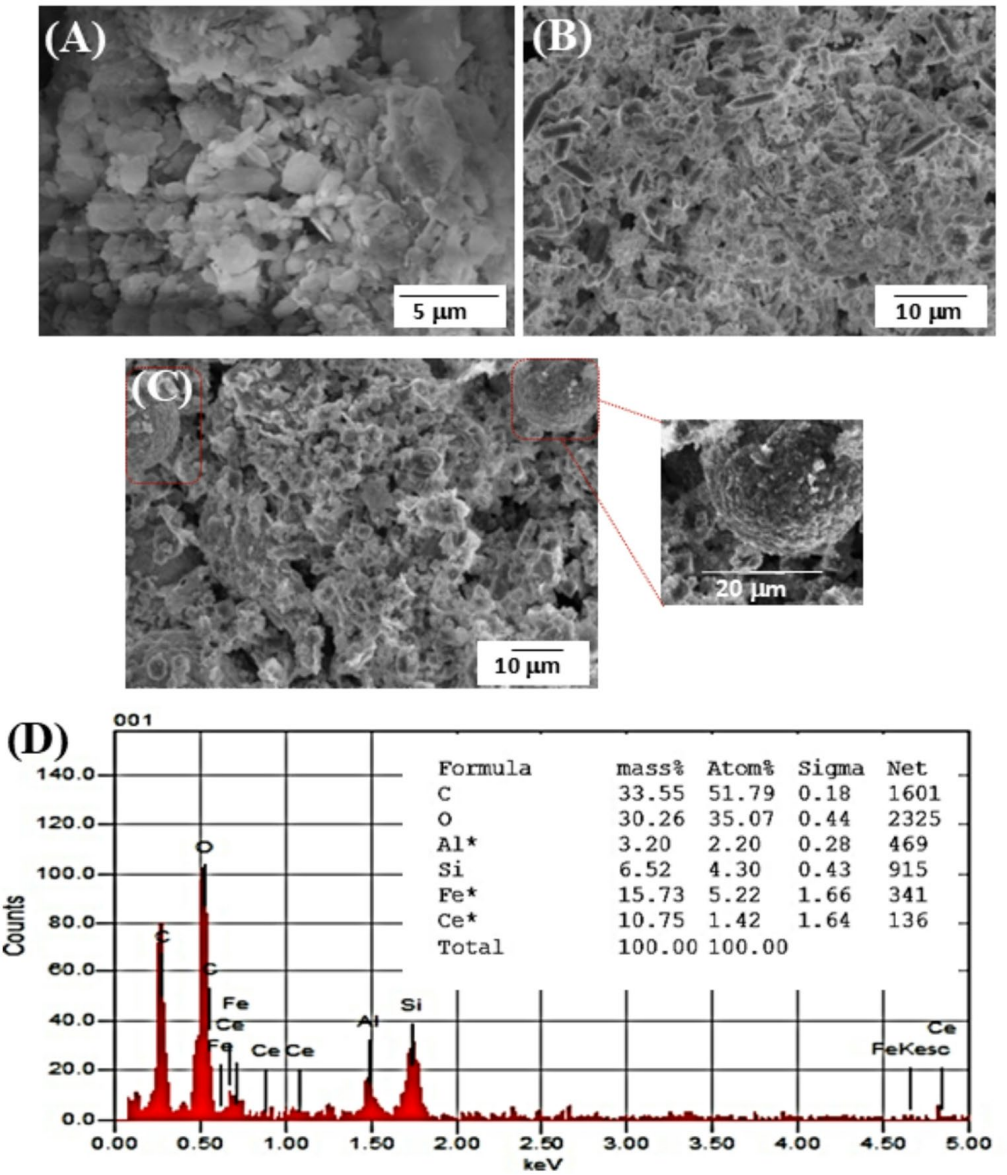
SEM images of (**A**) MMt_D2000_, **B** Ce/Fe-Tph., and (**C**) Ce/Fe-Tph./MMt_D2000_ Framework. **D** The typical EDX spectrum of the Ce/Fe-Tph./MMt_D2000_ framework (Inset; the EDX elemental mapping distribution of Fe, Ce, Si, Al, O, and C elements)

SEM and BET analyses were conducted to investigate the morphological characteristics and specific surface areas of the synthesized materials, as these properties significantly influence their performance. The SEM micrograph of MMt_D2000_ [Fig. 2A] revealed a layered, flake-like structure. In contrast, the SEM images of porous Ce/Fe-Tph. [Fig. 2B] exhibited an irregular morphology interspersed with uniform, pin-like nanorods approximately 333.0 nm in average diameter, as determined using the ImageJ program. Furthermore, the Ce/Fe-Tph./MMt_D2000_ Framework [Fig. 2C] displayed an irregular morphology combined with uniform spherical particles approximately 20 μm in diameter which corresponds to a yolk-shell morphology, without any observable MMt stack aggregates. The SEM-EDX analysis of the Ce/Fe-Tph./MMt_D2000_ framework showed clear signals for Fe, Ce, O, and C, confirming the formation of the MOF. Strong peaks for Al and Si also appeared, demonstrating the successful incorporation of MMt into the porous structure of the composite, as duplicated in [Fig. 2D].

As illustrated in [Fig. S_1_(A), (B)], nitrogen adsorption–desorption isotherms and BJH pore size distribution analyses were performed to evaluate the textural properties of the Ce/Fe-Tph./MMt_D2000_ framework nanocomposite. BET measurements indicated that the Ce/Fe-Tph./MMt_D2000_ framework possessed remarkably high specific surface area (1054.3 m²/g). Moreover, the isotherm exhibits a typical Type IV curve with a distinct H_3_-type hysteresis loop, indicating the presence of mesopores formed by slit-like pores or layered structures, which is consistent with the incorporation of montmorillonite. A sharp increase in adsorption volume at high relative pressures (P/P₀ ≈ 0.9–1.0) demonstrates capillary condensation within mesopores, supporting the measured high specific surface area. The BJH pore size distribution reveals a dominant pore width in the range of ~ 13–18 nm, further confirming mesoporosity. These characteristics indicate that the material possesses a highly porous structure. Also, the high BET specific surface area of the Ce/Fe-Tph./MMt_D2000_ framework is anticipated to enhance electron and ion transport, thereby improving the electrochemical performance of the material.

The Ce/Fe-Tph./MMt_D2000_ framework nanocomposite exhibits two distinct points of zero charge pH_PZC_ at approximately pH 4 and 11.8 as displayed in [Fig. S_2_] due to the presence of both inorganic (Ce^3+^, Fe^3+^, Al^3+^) and organic (BDC linker and Jefamine D_2000_) components. At low pH (~ 4), metal hydroxyl groups on the montmorillonite surface (such as ≡ Fe–OH and ≡ Al–OH) become protonated, forming positively charged species like ≡ Fe–OH₂⁺ and ≡ Al–OH₂⁺ of MMt [66]:

$$\begin{array}{c}\equiv Fe-OH+H^+\rightarrow\equiv Fe-OH^{2+}\\\equiv Al-OH+H^+\rightarrow\equiv Al-OH^{2+}\end{array}$$

Additionally, the benzene dicarboxylate (BDC) linker becomes partially protonated to HBDC, reducing its negative charge:

$$BDC^{2-}+H^+\rightarrow HBDC^-$$

These protonation reactions lead to a nearly neutral surface at pH 4. In contrast, at high pH (~ 11.8), surface hydroxyl groups on Ce and Fe sites are deprotonated, forming negatively charged species:

$$\begin{array}{c}\equiv Ce-OH\rightarrow\equiv Ce-O^-+H^+\\\equiv Fe-OH\rightarrow\equiv Fe-O^-+H^+\end{array}$$

The BDC linker becomes fully deprotonated to –COO⁻ [67], and Jefamine D_2000_, a polyetheramine, contributes –NH_2_ and ether groups that can also lose protons or interact with hydroxide, influencing surface charge distribution. These combined reactions result in a second point of zero charge around pH 11.8. The presence of two pH_PZC_ values reflects the heterogeneous surface behavior of the composite, enabling it to interact with both cationic and anionic species over a wide pH range, which is favorable for As^3+^ adsorption.

Based on its surface chemistry and electrochemical behavior, it is anticipated that the Ce/Fe-Tph./MMt_D2000_ framework will exhibit optimal conditions when operating at pH 12 for As³⁺ (predominantly HAsO₃^2^⁻ at this pH) detection via stripping cathodic voltammetry. Under such strongly alkaline conditions, surface groups like ≡ Fe–OH and ≡ Ce–OH are expected to undergo complete deprotonation, forming negatively charged ≡ Fe–O⁻ and ≡ Ce–O⁻ species:

$$\equiv Fe-OH\rightarrow\equiv Fe-O^-+H^+$$

This negative surface may enhance electrostatic attraction and facilitate adsorption of arsenite species such as HAsO₃²⁻ [68], the dominant form at high pH:

$$As{\left(OH\right)}_3\leftrightarrow HAsO_3^{2-}+2H^+$$

Concurrently, protonated interfering species like AlOH₂⁺ and HBDC become neutral or deprotonated, effectively minimizing non-specific background signals. Additionally, the presence of hydroxide ions facilitates ligand–OH⁻ coordination to metal centers, enhancing electron transfer and redox cycling essential for efficient arsenite reduction and reoxidation (As³⁺ + 3e⁻ ⇌ As⁰). These combined effects significantly improve the sensitivity and selectivity of arsenite detection, establishing pH 12 as the optimal operating condition.

### Characterization of the surface of stripping voltammetric sensors

#### Electroactive surface area and resistivity characteristics

**Fig. 3:**
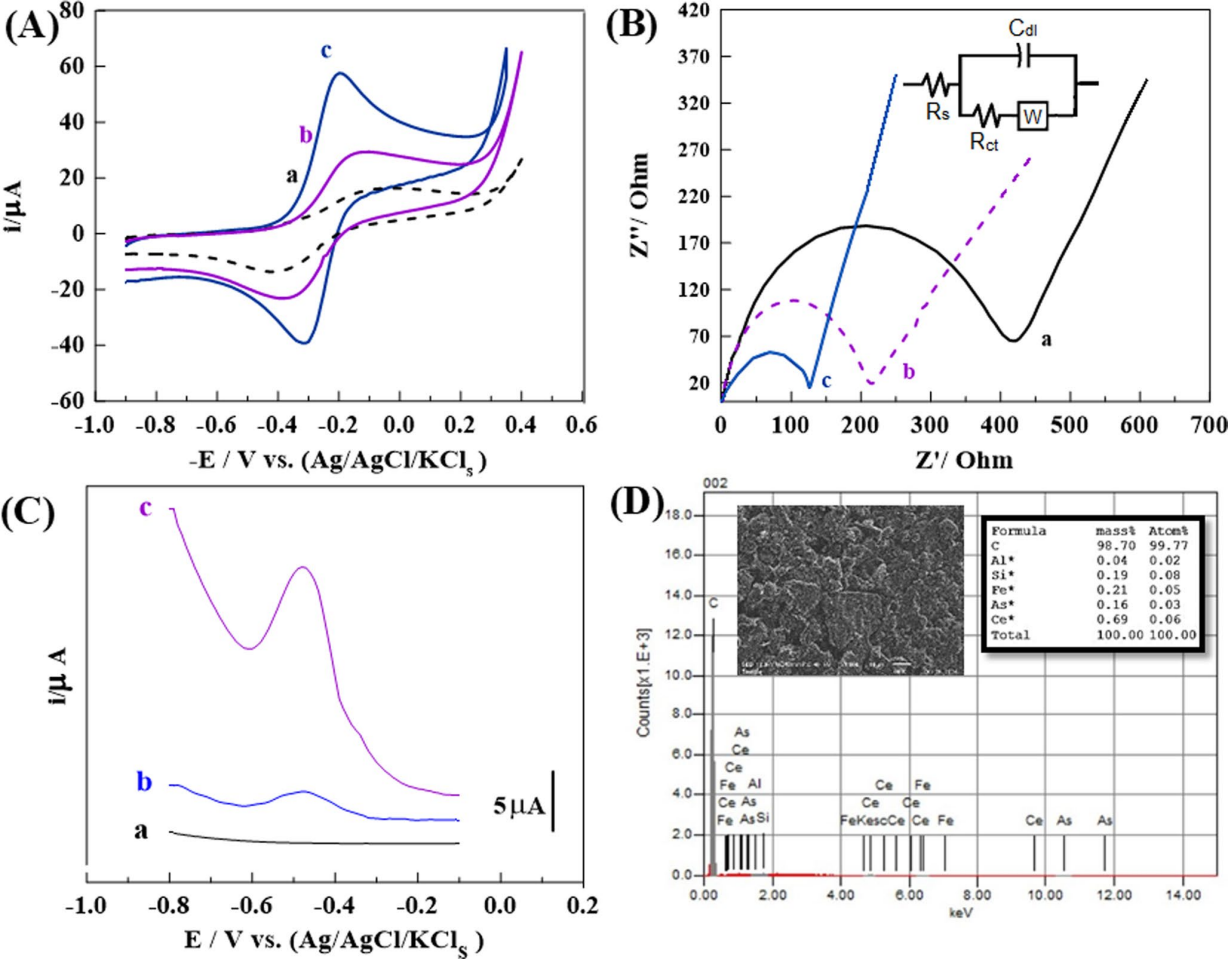
**A** CVs of 10 mM of K_3_[Fe(CN_6_)] in 0.1 M of KCl (***v*** = 50 mV·s^− 1^), and (**B**) Nyquist plots utilizing (a) BGPS, (b) 1.0% (Ce/Fe-Tph.) MGPS and (c) 1.0% (Ce/Fe-Tph./MMt_D2000_) MGPS (*n* = 3). **C** Voltammograms were obtained for 1.0nM As³⁺ ions at PBS (pH 12) using (a) BGPS, (b) 1.0% (Ce/Fe-Tph.) MGPS and (c) 1.0% (Ce/Fe-Tph./MMt_D2000_) MGPS (**t**_**acc**_=35 s, ***a*** = 25 mV, ***f*** = 90 H_z_ and Δ**E**_**s**_=10 mV), and (**D**) SEM-EDX elemental mass percent spectrum of 1.0% (Ce/Fe-Tph./MMt_D2000_) MGPS

To better understand the sensing mechanism of the modified stripping voltammetric sensors, the electroactive surface area of each synthesized sensor was evaluated. Cyclic voltammetry (CV) measurements were conducted using a 1.0 mM solution of K₃[Fe(CN)₆] in 0.1 M KCl as the redox probe, with a scan rate of 100 mV·s⁻¹. [Fig. 3 A] shows the CV profiles for the BGPS, 1.0% (Ce/Fe-Tph.) MGPS and 1.0% Ce/Fe-Tph./MMt_D2000_ MGPS. The CV curves displayed clear redox peaks, indicating a reversible electron transfer process for the [Fe(CN)_6_]^3-/4-^couple. Notably, the peak-to-peak separation (ΔE_p_) values for the unmodified and modified sensors were significantly lower, 210, and 100 mV for 1.0% (Ce/Fe-Tph.) MGPS, and 1.0% (Ce/Fe-Tph./MMt_D2000_) MGPS, respectively, compared to 360 mV for the BGPS. This decline in ΔEₚ indicates enhanced electron transfer kinetics, likely due to increased charge density and conductivity on the modified sensor surfaces. Furthermore, the voltammogram [Fig. 3 A] represents that voltammogram of 1.0% (Ce/Fe-Tph./MMt_D2000_) MGPS exhibited the largest peak current in comparison to 1.0% (Ce/Fe-Tph.) MGPS and BGPS, demonstrating their superior sensitivity. Among all, the 1.0% (Ce/Fe-Tph./MMt_D2000_) MGPS showed the most prominent redox response, attributed to its optimal balance of surface area and electrical conductivity from the Ce and Fe metal ions.

To quantify the electroactive surface area (A_s_), further CV experiments were conducted in 1.0 mM K_4_[Fe(CN)_6_] across a scan rate range of 50–500 mV·s⁻¹, and the resulting cathodic peak currents (I_p_) were plotted against the square root of the scan rate (ν½), as shown in [Fig.S_3_].Using the Randles–Sevcik equation [69], the A_s_ values were determined to be 0.065, 0.10, and 0.15 cm² for the BGPS and the 1.0% (Ce/Fe-Tph.), and 1.0% (Ce/Fe-Tph./MMt_D2000_) MGPS, respectively. Remarkably, the 1.0% (Ce/Fe-Tph./MMt_D2000_) MGPS demonstrated 2.5-fold increase in electroactive surface area compared to the unmodified sensor.

Electrochemical impedance spectroscopy (EIS) was also employed to investigate the charge transfer resistance (R_ct_) at the sensor–electrolyte interface. Nyquist plots were obtained in 0.1 M KCl containing 1.0 µM arsenite ions, with a frequency range from 0.1 H_z_ to 10,000 H_z_ [Fig. 3B]. The R_ct_ values derived from the plots were 420, 230, and 125 Ω for the BGPS, 1.0% (Ce/Fe-Tph.) MGPS and the 1.0% (Ce/Fe-Tph./MMt_D2000_) MGPS, respectively. In summary, the 1.0% (Ce/Fe-Tph./MMt_D2000_) MGPS exhibited the lowest resistance, indicating superior electron transfer capability and minimal impedance, further confirming its enhanced electrochemical performance. The attributes of the electrochemical system of the 1.0% (Ce/Fe-Tph./MMt_D2000_) MGPS are delineated by the equivalent circuit model of (R(C(RW))), as depicted in [Fig. 3B; inset]. This model consists of four components: the resistance of the bulk solution (R_s_) in series with the parallel combination of the double layer capacitance (C_dl_), charge-transfer resistance (R_ct_), and Warburg impedance (W).

#### The preliminary stripping voltammetry test of as-prepared sensors

The SW-AdCSV technique was utilized to initially detect 1.0nM of arsenite ions in PBS with a pH of 12, as shown in [Fig. 3 C]. The applied cumulative potential (E_acc_) and time (t_acc_) were − 0.1 V and 35 s, respectively. As shown in [Fig. 3 C], 1.0% (Ce/Fe-Tph.) and the 1.0% (Ce/Fe-Tph./MMt_D2000_) MGPSs displayed an reduction peak at −0.5 V, corresponding to the reduction of arsenite is reduced to AsH_3_ (arsine gas) [70]. In [Figs. 3C_b, c_], the MGPSs containing 1.0% Ce/Fe-Tph. and 1.0% Ce/Fe-Tph./MMt_D2000_ displayed distinct voltammetric peaks compared to the BCPS. This improvement is attributed to the porous structure of the Ce/Fe-Tph. material, which enhances electron transfer on the sensor surface. Moreover, the 1.0% (Ce/Fe-Tph./MMt_D2000_) MGPS exhibited the highest peak current (i_p_) for As³⁺ ions detection, surpassing that of the Ce/Fe-Tph.-based sensor. This enhanced response may be associated with the adsorption capacity of the neutral MMT structure [37, 66], as well as an increase in the electroactive surface area due to improved structural organization and distribution of active sites on the electrode surface [71]. Among the tested concentrations 0.5, 1.0, and 2.0% (w/w) of (Ce/Fe-Tph./MMt_D2000_) MGPSs, the 1.0% (w/w) MGPS showed the highest voltammetric current response, as illustrated in [Fig. S_4_]. Based on these results, the 1.0% (w/w) (Ce/Fe-Tph./MMt_D2000_) MGPS is considered highly suitable for electroanalytical applications.

Moreover, (SEM-EDX) was used to determine the elemental composition of the Ce/Fe-Tph./MMt_D2000_ framework, as shown in [Fig. 3D]. The analysis confirmed the adsorption of arsenite ions on the surface of the 1.0% (Ce/Fe-Tph./MMt_D2000_) MGPS. The EDX spectrum indicated the presence of carbon (C), aluminum (Al), silicon (Si), iron (Fe), arsenic (As), and cerium (Ce), with corresponding mass percentages of 98.70, 0.04, 0.19, 0.21, 0.16, and 0.69%, respectively.

#### Adsorption behavior of CMPA on modified sensors

**Fig. 4:**
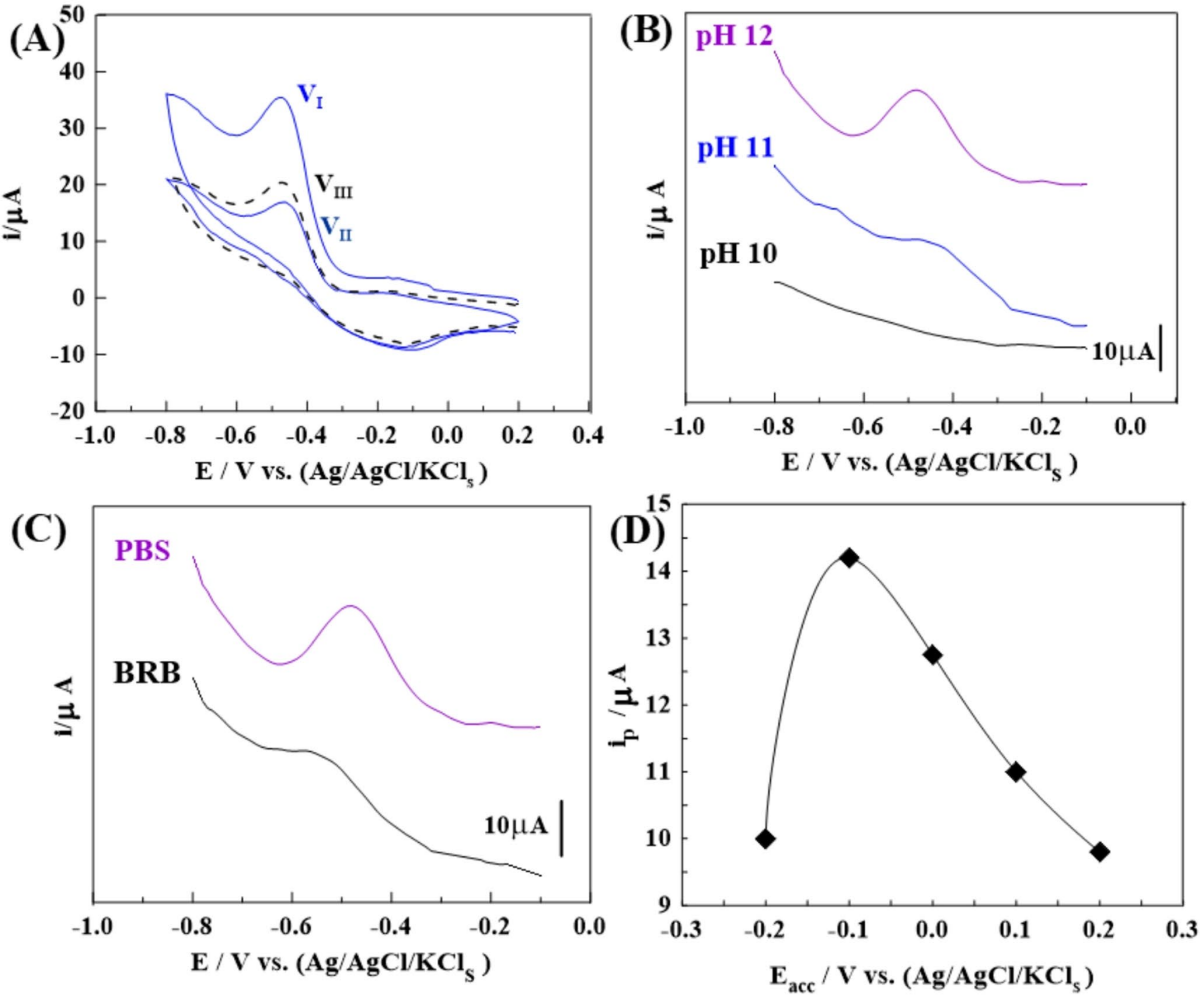
**(A)** CV voltammograms were recorded for 0.8 nM arsenite ions in (PBS; pH 12) using the 1.0% (Ce/Fe-Tph./MMt_D2000_) MGPS with t_acc_ of 50 s; 1 ^st^ cycle (V_I_), and 2^nd^ cycle (V_II_)), and without time (V_III_) at E_acc_= 0.2 V, and *v* = 100 mV.s^− 1^. Voltammograms were obtained for 1.0 nM arsenite ions in **(B)** different pH values, **(C)** different types of pH using the 1.0% (Ce/Fe-Tph./MMt_D2000_) MGPS (**t**_**acc**_=35s, ***a*** = 25 mV, ***f*** = 100 H_z_ and Δ**E**_**s**_=10 mV), and **(D)** Effect of changing of *E*_*acc*_ of 0.9 nM arsenite ions in (PBS buffer; pH 12) on the surface of 1.0% (Ce/Fe-Tph./MMt_D2000_) MGPS (*t*_*acc*_ = 35 s)

To evaluate the adsorption behavior of arsenite ions, CV experiments were performed using 1.0nM arsenite ions in PBS of pH 12. Measurements were carried upon the surface of the 1.0% (Ce/Fe-Tph./MMt_D2000_) MGPS **[**Fig. 4A**]**. The peak current (V_III_) was initially recorded under open circuit conditions, without employing adsorptive accumulation. Subsequently, voltammograms corresponding to the first cycle (V_I_) and second cycle (V_II_) were acquired following an adsorptive accumulation time of 50s. These results demonstrated the adsorption of As³⁺ ions onto the sensor surfaces, followed by their electrochemical detection.

#### Optimization of instrumental and stripping parameters

##### Influence of pH and buffer composition on electrochemical performance

The performance of arsenite ions detection through cathodic stripping voltammetric analysis is significantly influenced by the nature and concentration of the supporting electrolyte, as these factors govern the electrochemical interactions between the electroactive species and the sensor surface. To optimize these conditions, stripping voltammetric responses of 1.0nM As^3+^ ions were recorded using the 1.0% (Ce/Fe-Tph./MMt_D2000_) MGPS in PBS solutions with pH values ranging from 2 to 12. The accumulation parameters were maintained at E_acc_ = −0.1 V and t_acc_ = 35 s. Among the tested conditions, the PBS buffer at pH 12 yielded the most prominent and well-defined peak current response **[**Fig. 4B**]**, and was consequently selected as the optimal pH for arsenite ions detection. Subsequently, the SW-AdCSV peak current intensity of of 5.0 nM As^3+^ ions was evaluated utilizing the basic pH medium (pH ≈ 12) of BRB, and PBS buffers as figured out in **[**Fig. 4C**]**. The maximum and well-defined peak current intensity has been developed in PBS buffer (pH 12) attributable to its superior buffering capacity and ionic strength under alkaline conditions, which ensure stable electrochemical behavior conducive to arsenite ions reduction. The primary mechanism involves the transformation of arsenite (AsO₃³⁻) into elemental arsenic (As⁰) via the reaction:

$$AsO_3^{3-}+3H_2O+2e^-\rightarrow As^o+6OH^-$$

In this context, the nanocomposite-modified electrode plays a critical role, where Ce^3+^/Ce^4+^ and Fe^2+^/Fe^3+^ redox pairs accelerate electron transfer, montmorillonite layers support effective arsenite adsorption, and the Jeffamine D_2000_ polymer matrix enhances interaction and dispersion. The high conductivity of PBS and favorable phosphate interactions further stabilize intermediate species and promote efficient electron flow, yielding sharper and more reproducible stripping voltammetric signals than the less stable B–R buffer at the same pH. Therefore, the PBS buffer (pH 12) was chosen as a supporting electrolyte for the subsequent studies.

##### Influence of SW instrumental and accumulation parameters on the electrochemical performance

Furthermore, by varying the parameters of frequency (***f***) from (10–100 Hz), pulse height (***a***) from (5 to 25 mV) and scan increment (**ΔE**_**s**_) from (2–10 mV) **[Figs.S**_**5**_**A**,** B**,** C]**, the optimal instrumental conditions for the electrochemical detection of As^3+^ ions using the 1.0% (Ce/Fe-Tph./MMt_D2000_) MGPS were obtained at 100 H_z_, 25 mV, 10 mV, respectively.

Additionally, the adsorption-driven stripping step was found to significantly improve detection sensitivity. To evaluate accumulation parameters, the effect of varying accumulation potential (E_acc_) from (0.2 to −0.2 V) on the cathodic peak current intensity of 1.0 nM As^3+^ ions in PBS at pH 12 was tested. As shown in **[**Fig. 4D**]**, the highest peak current was observed at E_acc_ = −0.1 V with an accumulation time (t_acc_) of 35 s. Moreover, the effect of accumulation time (t_acc_) on the cathodic peak current intensity of As^3+^ ions was systematically investigated at concentration of 0.9 nM using the 1.0% (Ce/Fe-Tph./MMt_D2000_) MGPS in PBS buffer at pH 12. As shown in [Fig.S_5_D], the peak current increased linearly with t_acc_ up to 70 s, followed by a decline due to sensor surface saturation with As^3+^ ions. Based on these results, the optimal accumulation conditions were established as E_acc_ = −0.1 V and t_acc_ = 60 s, ensuring enhanced sensitivity and reproducibility for subsequent measurements.

#### 3.2.4. Method validation

##### Linearity range and limit of detection (LOD)

**Fig. 5:**
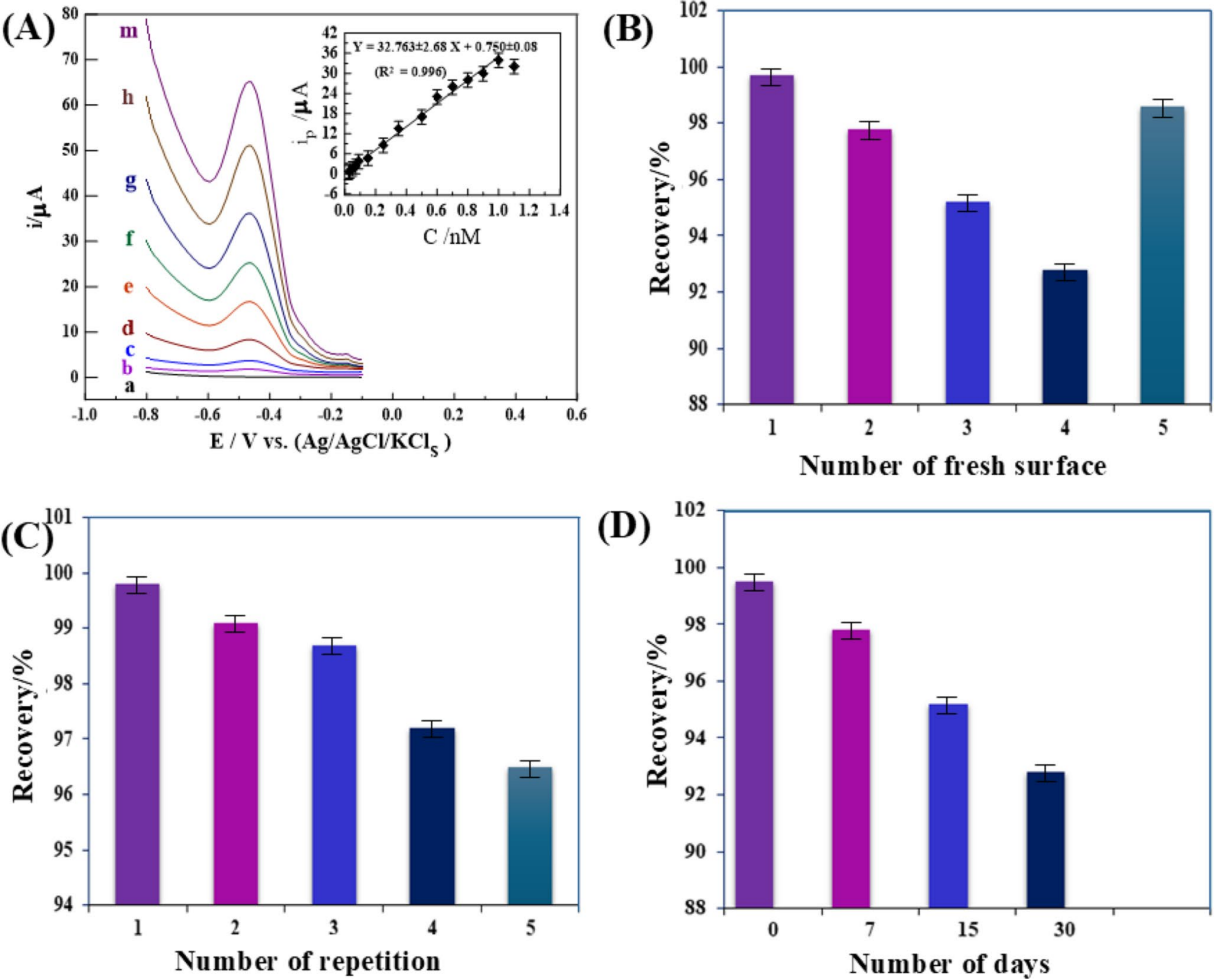
**A** SW–AdCV voltammograms of different amounts of As^3+^ ions in ***pH*** 12 on 1.0% (Ce/Fe-Tph./MMt_D2000_) MGPS (***E***_***acc***_= −0.1 V, ***t***_**acc**_= 60 s, ***Δ*E**_***s***_= 10 mV, ***f*** = 90 H_z_, and ***a*** = 25 mV) in bulk form: (a) baseline, (b) 0.02, (c) 0.04, (d) 0.08, (e) 0.2, (f) 0.4, (g) 0.6, (h) 0.8, and (m) 1.0 nM (inset: its corresponding plot (*n* = 3)). Histogram of **(B)** reproducibility, **C** repeatability, and (**D**) stability of 0.3 nM (0.0225 ppb) As^3+^ ions on the surface of 1.0% (Ce/Fe-Tph./MMt_D2000_) MGPS

Using optimal analytical parameters, the SW-AdCSV process was validated by assessing various analytical aspects, including accuracy, precision, limit of detection (LOD), and the linearity range of the calibration curve. As depicted in [Fig. 5 A], the calibration voltammograms and plots for As^3+^ ions determination in PBS buffer at pH 12 on the surface of a 1.0% (Ce/Fe-Tph./MMt_D2000_) MGPS were determined, with linearity ranges (LR) of 0.02 to 1.0nM (0.0015–0.0749 ppb), yielding sensitivity values (S/*N* = 3) of 32.7 µA.nM^− 1^ (436.68 µA/ppb). The calibration plot was expressed by the following linear regression equations: i_p/µA_= (32.763 ± 2.68) C_As_^+3^_/nM_- (0.750 ± 0.08) (R^2^ = 0.996), with a LOD of approximately 0.006 nM (0.00045 ppb). Several electroanalytical techniques and sensors have been developed for As^3+^ ions detection in various fluids, as summarized in [Table 1].

The 1.0% (Ce/Fe-Tph./MMt_D2000_) MGPS developed in this study demonstrates superior analytical performance compared to previously reported As^3+^ ions sensors. For example, Dahake et al. [24] and Pereira et al. [25] observed interference from other metal ions and limited linear detection ranges. Sensors developed by Guo et al. [26] and Ismail et al. [27] reported higher detection limits and moderate sensitivity, while Sullivan et al. [28] and Rashmi et al. [29] provided useful data but lacked validation in complex environmental samples.

By comparison, the sensor introduced in this work achieves an exceptionally low detection limit (0.00045 ppb) with an extended linear range (0.0015–0.0749 ppb), far exceeding the capabilities of earlier methods [Table 1]. Its sensitivity (436.68 µA/ppb) is also markedly higher, allowing precise quantification at trace levels. Most significantly, this method exhibits strong resistance to interference from most of the probable interfering heavy metal ions due to the high alkalinity of the optimal supporting electrolyte (pH 12) and performs effectively in untreated bulk and soil samples. These advantages position this sensor as a promising candidate for accurate and sustainable As^3+^ monitoring in real environmental contexts.

**Table 1:** Overview of electroanalytical sensors and performance parameters utilized in the detection of arsenite ions

Developed Sensor	Technique	LR ppb	Sensitivity µA/ppb	LOD ppb	Ref.
Citrate-stabilized Au NP-modified GCE	DPV - SWV	Not stated	Not stated	0.025	[24]
Thoria NP–carbon paste	ASV	0–180	0.54	0.1	[25]
rGO-Au_nano_/GCE	SWASV	1.0–50	1.521	0.08	[26]
Silica NP-modified SPCE	SW-AdASV	10–50	0.327	6.2	[27]
Au NP-modified SPCE	SW-AdSV	5–30	0.1007	16.73	[28]
rGO–CeO_2_	SW-AdSV	10–500	2.13	0.93	[29]
1.0% (Ce/Fe-Tph./MMt_D2000_) MGPS	SW-AdCSV	0.0015–0.0749 (0.02–1.0.02.0 nM)	436.68	0.00045 (Bulk) (0.006 nM)	**This work**

##### Assessment of the analytical validity of the modified sensor

Under optimized experimental conditions, the reliability and repeatability of the sensor were thoroughly assessed using both intra-day and inter-day analysis. Intra-day precision was evaluated by recording SW-AdCS voltammograms of 0.3 nM (0.0225 ppb) As^3+^ ions using five freshly prepared 1.0% (Ce/Fe-Tph./MMt_D2000_) MGPSs measured in parallel within a single day. Inter-day performance was examined across five consecutive days using the same approach. The mean recovery and relative standard deviation (R% ± RSD) were calculated as 98.26% ± 1.39 for intra-day analysis [Fig. 5B] and 97.48% ± 1.22 for inter-day analysis [Fig. 5 C], indicating high reliability and excellent reproducibility of the sensor response.

To further evaluate the long-term stability of the sensor, the modified electrodes were stored in ambient air at room temperature (RT) and tested every 7 days over a 30-day period (*n* = 3). The sensor retained 95.20% of its initial activity after 15 days and 92.8% after 30 days, demonstrating excellent signal retention [Fig. 5D]. As summarized in **[**Table 2**]**, these results confirm that the sensor possesses robust reliability, consistent repeatability, and substantial storage stability primarily attributed to the chemical integrity of the Ce/Fe-Tph./MMt_D2000_modifier incorporated into the sensing platform.

**Table 2:** The intra and inter-day assay of 0.3 nM (0.0225 ppb) arsenite ions in its pure form utilizing the SW-AdCSV method:

	**C**_Taken_**(nM)**	**C**_Found_**(nM) ± SD**	**Recovery ± Precision** **(R % ± RSD %)**	**Relative Error** **Er (%)**
***Intra-day analysis***	0.3 (0.0225 ppb)	0.295 ± 0.05	98.26 ± 1.39	-1.74
***Inter-day analysis***	0.3 (0.0225 ppb)	0.292 ± 0.054	97.48 ± 1.22	-2.52
***Stability***_(n=3)_*				
***15 days***	0.3(0.0225 ppb)	0.286 ± 0.060	95.20 ± 2.24	-4.80
***30 days***		0.278 ± 0.076	92.30 ± 3.40	-7.20

* (***n***= 3) means three measurements.

#### Selectivity

**Fig. 6:**
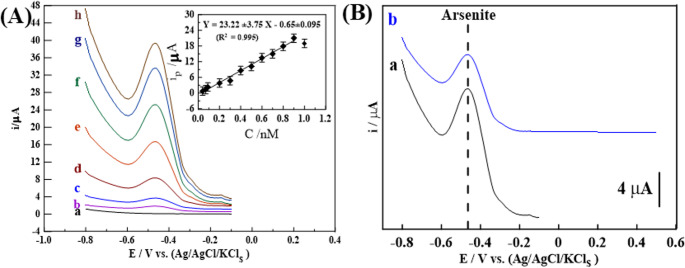
**(A)** SW–AdCV voltammograms of different amounts of As^3+^ ions in ***pH*** 12 on 1.0% (Ce/Fe-Tph./MMt_D2000_) MGPS (***E***_***acc***_= −0.1 V, ***t***_**acc**_= 60 s, ***Δ*E**_***s***_= 10 mV, ***f*** = 90 H_z_, and ***a*** = 25 mV)in soil_1_ form: (a) baseline, (b) 0.04, (c) 0.07, (d) 0.2, (e) 0.4, (f) 0.6, (g) 0.8, and (h) 0.9 nM (inset: its corresponding plot (*n* = 3)). **(B)** SW–AdAV voltammograms for **(a)** 0.3 nM (0.0225 ppb) As^3+^ ions, **(b)** 0.3 nM (0.0225 ppb) As^3+^ ions mixed with **Mix**_**1**_ in pH 12 on the surface of 1.0% (Ce/Fe-Tph./MMt_D2000_) MGPS

The anti-interference performance (selectivity) of the 1.0% Ce/Fe-Tph./MMt_D2000_ modified MGPS was investigated by the addition of common interfering speciesin water andsoil samples, as displayed in **[**Fig. 6B**]**. Whereas the ***i***_**p**_ voltammogram of 0.3 nM (0.0225 ppb) As^3+^ ions was evaluated after addition of 300 nM (~ 1000-fold) of major cations and anions such as (Mix_1_: Ca^+ 2^, Mg^+ 2^, Hg^2+^, Pb^2+^,Al^3+^, Zn^2+^, Ni^2+^, Cr^3+^, Cu^2+^, Na^+^, K^+^, Cl^−^, SO_4_^2−^, NO_3_^−^ and HCO_3_^−^) at pH 12. In this pH medium, The As³ is the most likely to exhibit a distinct peak in SW-AdCSV due to its speciation as HAsO_3_²⁻, which adsorbs onto negatively charged surface of the modified sensor and undergoes efficient reduction to As⁰. Most trace and ultra-trace metal ions, including Cu^2+^, Cd^2+^, Co^2+^, Fe^2+^, Zn^2+^, Ni^2+^, Mn^2+^, Hg^2+^, Pb^2+^, Al^3+^, Cr^3+^ and Fe^3+^, either form insoluble hydroxides at elevated pH levels or necessitate anodic stripping conditions for detection, rendering them inappropriate for SW-AdCSV detection [72, 73]. Additionally, SW-AdCSV relies on adsorption and reduction of analytes that remain soluble and electroactive at the working sensor, which is not the case for most of these metals at pH 12 due to hydrolysis, precipitation, or lack of redox activity in this medium. This is confirmed by the fact that there are no extra peaks corresponding to any of the investigated interfering ions applying SW-AdCSV at 1.0% Ce/Fe-ph./MMt_D2000_ modified MGPS under the optimal conditions.

#### Application in environmental real samples

Under optimized analytical conditions, SW-AdCS voltammograms and the corresponding calibration plot were obtained for As^3+^ ions detection in PBS (pH 12). The analysis was performed using a 1.0% Ce/Fe-Tph./MMt_D2000_ modified MGPS surface, with a spiked soil sample (Soil_1_) added directly, thus eliminating any sample pre-treatment. As shown in **[**Fig. 6A**]**, the linear response (LR) ranged from 0.04 to 0.9 nM with a sensitivity of 23.22 µA·nM⁻¹. The calibration curve followed a linear regression equation: i_p(µA)_ = (23.22 ± 3.75)·C_arsenite(nM)_ + (0.65 ± 0.095) with a high correlation coefficient (R² = 0.995) and a limit of detection (LOD) of approximately 0.012 nM. All investigated real samples (brackish water, sea water and two soil samples) were analyzed applying the proposed method and no As^3+^ ions were detected. Therefore, two concentration levels of 0.07 (0.00524 ppb) and 0.5 nM (0.03746 ppb) As^3+^ ions were spiked in each of the real sample and measured. The sensor exhibited satisfactory recovery (R%) ± relative standard deviation (RSD%) as detailed in [Table 3], indicating minimal interference from water and soil constituents. These results suggest that the sensor provides highly accurate (RE%) and reliable detection of As^3+^ ions, even in complex environmental matrices.

**Table 3:** Detection of As^3+^ ions carried out in different real samples (*n* = 3)

***Sample***	C_Added_(nM)	C_Found_(nM) ± SD		*R* % ± RSD%		RE%
***Brackish Water***	0.07 0.5	0.0695 ± 0.004 0.4958 ± 0.087		99.29 ± 1.57 99.16 ± 1.10		−0.71 −0.84
***Sea Water***	0.070.5	0.0690 ± 0.0080.4975 ± 0.092		98.57 ± 1.7699.50 ± 2.12		−1.43−0.5
***Soil***_***2***_	0.070.5	0.0689 ± 0.0020.4946 ± 0.075		98.43 ± 2.0298.92 ± 3.46		−1.57−1.08
***Soil***_***3***_	0.07 0.5	0.0709 ± 0.007 0.5036 ± 0.045		101.3 ± 3.56 100.7 ± 1.07		1.30 0.70

## Conclusion

A yolk-shell (Ce/Fe) Terephthalate (Ce/Fe-Tph.) framework intercalated with organoclay (MMt_D2000_) nanolayers was successfully synthesized via a one-step hydrothermal method. The yolk-shell Ce/Fe-Tph./MMt_D2000_ framework nanocomposite was synthesized using a solvothermal method, resulting excellent crystallinity and a high surface area of 1054.3 m².g⁻¹. This framework nanocomposite was incorporated into a graphite paste sensor for the detection of As^3+^ ions in soil samples using SW-AdCSV technique. Electrochemical characterization by CV and EIS revealed enhanced electroactive surface area, faster electron transfer, and stronger adsorption capacity compared to the BGPS. The developed sensor represented exceptional sensitivity and stability compared to the previously reported electrochemical sensors, coupled with exceptional selectivity for As^3+^ ions, even in the presence of elevated concentrations of other heavy metals. The developed sensor was successfully applied for detecting As^3+^ ions in water and soil real samples without requiring complex sample pretreatment.

## Supplementary Information

Below is the link to the electronic supplementary material.

Supplementary Material 1 (DOCX 438 KB)

## Data Availability

All data used or produced in this study are presented within this work.

## References

[1] Duffus JH (2002) Heavy metals" a meaningless term?(IUPAC Technical Report). Pure Appl Chem 74:793–807

[2] Aragay G, Pons J, Merkoçi A (2011) Recent trends in macro-, micro-, and nanomaterial-based tools and strategies for heavy-metal detection. Chem Rev 111:3433–3458 21395328 10.1021/cr100383r

[3] Martin S, Griswold W (2009) Human health effects of heavy metals, Environmental Science and Technology briefs for citizens, 15 1–6

[4] Cui L, Wu J, Ju H (2016) Label-free signal-on aptasensor for sensitive electrochemical detection of arsenite. Biosens Bioelectron 79:861–865 26785310 10.1016/j.bios.2016.01.010

[5] Podgorski J, Berg M (2020) Global threat of arsenic in groundwater. Science 368:845–850 32439786 10.1126/science.aba1510

[6] Farzan SF, Karagas MR, Chen Y (2013) Utero and early life arsenic exposure in relation to long-term health and disease. Toxicol Appl Pharmacol 272:384–390 23859881 10.1016/j.taap.2013.06.030 PMC3783578

[7] Ravenscroft P, Brammer H, Richards K (2011) Arsenic pollution: a global synthesis. Wiley

[8] Bhat A, Hara TO, Tian F, Singh B (2023) Review of analytical techniques for arsenic detection and determination in drinking water. Environ Science: Adv 2:171–195

[9] Álvarez-Llamas G, del Rosario Fernández de laCampa Ma, Sanz-Medel A (2005) ICP-MS for specific detection in capillary electrophoresis. TRAC Trends Anal Chem 24:28–36

[10] Feng Y-L, Chen H-Y, Tian L-C, Narasaki H (1998) Off-line separation and determination of inorganic arsenic species in natural water by high resolution inductively coupled plasma mass spectrometry with hydride generation combined with reaction of arsenic (V) and L-cysteine. Anal Chim Acta 375:167–175

[11] Macedo SM, de Jesus RM, Garcia KS, Hatje V, Queiroz AF, Ferreira SL (2009) Determination of total arsenic and arsenic (III) in phosphate fertilizers and phosphate rocks by HG-AAS after multivariate optimization based on Box-Behnken design. Talanta 80:974–979 19836581 10.1016/j.talanta.2009.08.025

[12] Ma J, Sengupta MK, Yuan D, Dasgupta PK (2014) Speciation and detection of arsenic in aqueous samples: a review of recent progress in non-atomic spectrometric methods. Anal Chim Acta 831:1–23 24861967 10.1016/j.aca.2014.04.029

[13] Zhang N, Fu N, Fang Z, Feng Y, Ke L (2011) Simultaneous multi-channel hydride generation atomic fluorescence spectrometry determination of arsenic, bismuth, tellurium and selenium in tea leaves. Food Chem 124:1185–1188

[14] Bonthula S, Devarajan S, Maurya MR, Al-Maadeed S, Maalej R, Chaari MZ, Sadasivuni KK (2024) Advancing rapid arsenic (III) detection through device-integrated colorimetry. Chem Afr 7:4381–4391

[15] Jia X, Gong D, Wang J, Huang F, Duan T, Zhang X (2016) Arsenic speciation in environmental waters by a new specific phosphine modified polymer microsphere preconcentration and HPLC–ICP-MS determination. Talanta 160:437–443 27591635 10.1016/j.talanta.2016.07.050

[16] Manikandan R, Rajarathinam T, Jayaraman S, Jang H-G, Yoon J-H, Lee J, Paik H-j, Chang S-C (2024) Recent advances in miniaturized electrochemical analyzers for hazardous heavy metal sensing in environmental samples. Coord Chem Rev 499:215487

[17] Manikandan R, Yoon J-H, Chang S-C (2023) Emerging trends in nanostructured materials-coated screen printed electrodes for the electrochemical detection of hazardous heavy metals in environmental matrices. Chemosphere 344:140231 37775053 10.1016/j.chemosphere.2023.140231

[18] Manikandan R, Mani SP, Selvan KS, Yoon J-H, Chang S-C (2023) Fabrication of S and O-incorporated graphitic carbon nitride linked poly (1, 3, 4-thiadiazole-2, 5-dithiol) film for selective sensing of Hg2 + ions in water, fish, and crab samples. Food Chem 425:136483 37269636 10.1016/j.foodchem.2023.136483

[19] Pandey S, Mishra S (2025) A review of sensing technologies for arsenic detection in drinking water. Int J Environ Sci Technol 22:2809–2832

[20] Altama AK, Sriram B, Elanthamilan E, Chu JP, Liao Y-L, Wang S-F (2024) Hierarchical graphene oxide/nano-pyramidal stainless-steel on nickel foam substrate: a flexible electrochemical sensor for arsenic compound detection. J Environ Chem Eng 12:114179

[21] Cavicchioli A, La-Scalea MA, Gutz IG (2004) Analysis and speciation of traces of arsenic in environmental, food and industrial samples by voltammetry: a review. Electroanalysis 16:697–711

[22] Hrapovic S, Liu Y, Luong JH (2007) Reusable platinum nanoparticle modified Boron doped diamond microelectrodes for oxidative determination of arsenite. Anal Chem 79:500–507 17222013 10.1021/ac061528a

[23] Hamid Kargari S, Ahour F, Mahmoudian M (2023) An electrochemical sensor for the detection of arsenic using nanocomposite-modified electrode. Sci Rep 13:8816 37258602 10.1038/s41598-023-36103-6 PMC10232514

[24] Giacomino A, Abollino O, Lazzara M, Malandrino M, Mentasti E (2011) Determination of as (III) by anodic stripping voltammetry using a lateral gold electrode: experimental conditions, electron transfer and monitoring of electrode surface. Talanta 83:1428–1435 21238732 10.1016/j.talanta.2010.11.033

[25] Pereira F, Vázquez M, Debán L, Aller A (2016) Inorganic arsenic speciation by differential pulse anodic stripping voltammetry using thoria nanoparticles-carbon paste electrodes. Talanta 152:211–218 26992513 10.1016/j.talanta.2016.02.011

[26] Zhao G, Liu G (2018) Electrochemical deposition of gold nanoparticles on reduced graphene oxide by fast scan cyclic voltammetry for the sensitive determination of As (III). Nanomaterials 9:41 30597942 10.3390/nano9010041 PMC6359602

[27] Ismail S, Yusof NA, Abdullah J, Abd SF, Rahman (2020) Electrochemical detection of arsenite using a silica nanoparticles-modified screen-printed carbon electrode. Materials 13:3168 32708531 10.3390/ma13143168 PMC7412229

[28] Sullivan C, Lu D, Senecal A, Kurup P (2021) Voltammetric detection of arsenic (III) using gold nanoparticles modified carbon screen printed electrodes: application for facile and rapid analysis in commercial apple juice. Food Chem 352:129327 33690077 10.1016/j.foodchem.2021.129327

[29] Dahake RV, Bansiwal A (2022) Highly sensitive detection of arsenic in groundwater by paper-based electrochemical sensor modified with earth-abundant material. Groundw Sustain Dev 19:100855

[30] Tripathy RK, Samantara AK, Behera J (2019) A cobalt metal–organic framework (Co-MOF): a bi-functional electro active material for the oxygen evolution and reduction reaction. Dalton Trans 48:10557–10564 31215575 10.1039/c9dt01730e

[31] Ambaye AD, Kefeni KK, Kebede TG, Ntsendwana B, Mishra SB, Nxumalo EN (2022) Cu-MOF/N-doped GO nanocomposites modified screen-printed carbon electrode towards detection of 4-nitrophenol. J Electroanal Chem 919:116542

[32] Ambaye AD, Muchindu M, Jijana A, Mishra S, Nxumalo E (2023) Screen-printed electrode system based on carbon black/copper-organic framework hybrid nanocomposites for the electrochemical detection of nitrite. Mater Today Commun 35:105567

[33] Ambaye AD, Kebede TG, Ntsendwana B, Nxumalo EN (2023) Fe-MOF derived graphitic carbon nitride nanocomposites as novel electrode materials for the electrochemical sensing of 2, 4-dichlorophenol in wastewater. Synth Met 299:117452

[34] Elfiky M, Beltagi AM, Abuzalat O (2024) Adsorptive stripping voltammetric sensor based on Cd zeolitic imidazole framework-67 for electrochemical detection of sarin simulant. Microchim Acta 191:80 10.1007/s00604-023-06112-3 PMC10774163 38190052

[35] Elfiky M, Beltagi AM, Abuzalat O (2021) Selective modified stripping voltammetric sensor based on Ce-1, 4-benzenedicarboxylic metal–organic frameworks porous nanoparticles for picomolar detection of curcumin. J Electroanal Chem 898:115606

[36] Chen J-Y, Weng Y-X, Han Y-H, Ye R-H, Huang D-H, Safety E (2024) A novel pencil graphite electrode modified with an iron-based conductive metal-organic framework exhibited good ability in simultaneous sensing bisphenol A and bisphenol. Ecotoxicology and Environmental Safety 272:116065 10.1016/j.ecoenv.2024.116065 38330872

[37] Elfiky M, Abdo Mm, Darwesh M, Salahuddin N (2025) Ultra-sensitive detection of 4-chloro-2-methylphenoxyacetic acid herbicide using a porous Co-1, 4-benzenedicarboxylate/montmorillonite nanocomposite sensor. Microchim Acta 192:1–14 10.1007/s00604-024-06765-8 PMC11668838 39718606

[38] Atzori C, Ethiraj J, Colombo V, Bonino F, Bordiga S (2020) Adsorption properties of Ce5 (BDC) 7.5 (DMF) 4 MOF. Inorganics 8:9

[39] Sangeetha S, Krishnamurthy G (2020) Electrochemical and photocatalytic applications of Ce-MOF. Bull Mater Sci 43:1–10

[40] Maksimchuk NV, Zalomaeva OV, Skobelev IY, Kovalenko KA, Fedin VP, Kholdeeva OA (2012) Metal–organic frameworks of the MIL-101 family as heterogeneous single-site catalysts. Proc R Soc Lond A Math Phys Eng Sci 468:2017–2034

[41] Skobelev IY, Sorokin AB, Kovalenko KA, Fedin VP, Kholdeeva OA (2013) Solvent-free allylic oxidation of alkenes with O2 mediated by Fe-and Cr-MIL-101. J Catal 298:61–69

[42] Song J, Huang M, Lin X, Li SFY, Jiang N, Liu Y, Guo H, Li Y (2022) Novel Fe-based metal–organic framework (MOF) modified carbon nanofiber as a highly selective and sensitive electrochemical sensor for tetracycline detection. Chem Eng J 427:130913

[43] Janjani P, Bhardwaj U, Gupta R, Kushwaha HS (2022) Bimetallic Mn/Fe MOF modified screen-printed electrodes for non-enzymatic electrochemical sensing of organophosphate. Anal Chim Acta 1202:339676 35341509 10.1016/j.aca.2022.339676

[44] Li Z, Liu X, Jin W, Hu Q, Zhao Y (2019) Adsorption behavior of arsenicals on MIL-101 (Fe): the role of arsenic chemical structures. J Colloid Interface Sci 554:692–704 31352244 10.1016/j.jcis.2019.07.046

[45] Ramezani M-A, Najafi M, Karimi-Harandi M-H (2024) Highly sensitive determination of trace arsenic (III) onto carbon paste electrode modified with graphitic carbon nitride decorated Fe-MOF. Food Chem 458:140296 38959806 10.1016/j.foodchem.2024.140296

[46] Choudhary RB, Ansari S, Majumder M (2021) Recent advances on redox active composites of metal-organic framework and conducting polymers as pseudocapacitor electrode material. Renew Sustain Energy Rev 145:110854

[47] Xiao P, Zhu G, Shang X, Hu B, Zhang B, Tang Z, Yang J, Liu J (2022) An Fe-MOF/MXene-based ultra-sensitive electrochemical sensor for arsenic (III) measurement. J Electroanal Chem 916:116382

[48] Elfiky M, Salahuddin N, Matsuda A (2020) Green fabrication of 3D hierarchical blossom-like hybrid of peeled montmorillonite-ZnO for in-vitro electrochemical sensing of diltiazem hydrochloride drug. Materials Science and Engineering: C 111:110773 10.1016/j.msec.2020.110773 32279745

[49] Alizadeh A, Asghar S, Roudgar-Amoli M, Shariatinia Z, Protection E (2023) Water remediation using activated montmorillonite/metal-organic framework nanocomposites: response surface methodology, kinetic, and thermodynamic studies. Process Safety and Environmental Protection 177:507–529

[50] Wang J (2006) Analytical electrochemistry. Wiley-Vch

[51] Zhou A, Dou Y, Zhou J, Li JR (2020) Rational localization of metal nanoparticles in yolk–shell MOFs for enhancing catalytic performance in selective hydrogenation of cinnamaldehyde. ChemSusChem 13:205–211 31556474 10.1002/cssc.201902272

[52] Xu Z, Salem A, Chen Z, Zhang W, Chen J, Wang Z, Sun Q, Yin D (2008) Pb-210 and Cs-137 distribution in Burullus lagoon sediments of Nile river delta, Egypt: sedimentation rate after Aswan High Dam. Frontiers of Earth Science in China 2:434–438

[53] Elfiky M, Kumar R, Beltagi A (2022) Anthropogenic greenhouse CO<Subscript>2</Subscript> gas sensor based on glassy carbon modified with organoclay/polypyrrole-alginate nanocomposites in brackish water and seawater. J Electroanal Chem 926:116926

[54] Shahmahdi N, Dehghanzadeh R, Aslani H, Shokouhi SB (2020) Performance evaluation of waste iron shavings (Fe0) for catalytic ozonation in removal of sulfamethoxazole from municipal wastewater treatment plant effluent in a batch mode pilot plant. Chem Eng J 383:123093

[55] Wang T, Zhang J, Song Y, Liu Z, Ding H, Zhao C, Wang P (2021) Role of micro-size zero valence iron as particle electrodes in a three-dimensional heterogeneous electro-ozonation process for nitrobenzene degradation. Chemosphere 276:130264 34088105 10.1016/j.chemosphere.2021.130264

[56] Yue X, Liu Z, Zhang Q, Li X, Hao F, Wei J, Guo W (2016) Oxidative degradation of Rhodamine B in aqueous solution using Fe/PANI nanoparticles in the presence of AQS serving as an electron shuttle. Desalin Water Treat 57:15190–15199

[57] Yuvakkumar R, Elango V, Rajendran V, Kannan N (2011) Preparation and characterization of zero valent iron nanoparticles. Dig J Nanomater Biostruct 6:1771–1776

[58] Xu K, Li K, Tu D, Zhong T, Xie C (2014) Reinforcement on the mechanical-, thermal‐, and water‐resistance properties of the wood flour/chitosan/poly (vinyl chloride) composites by physical and chemical modification. J Appl Polym Sci. 10.1002/app.4075724994939

[59] Qiao M, Wu S, Ran Q, Shen J (2010) Preparation and properties of PU/MCMMT nanocomposites. Polym Adv Technol 21:296–299

[60] Zhang B, Zhang T, Zhang Z, Xie M (2019) Hydrothermal synthesis of a graphene/magnetite/montmorillonite nanocomposite and its ultrasonically assisted methylene blue adsorption. J Mater Sci 54:11037–11055

[61] Hadjltaief HB, Galvez ME, Zina MB, Costa PD (2014) TiO_2_/clay as a heterogeneous catalyst in photocatalytic/photochemical oxidation of anionic reactive blue 19. Arab J Chem

[62] Salahuddin N, Kenawy ER, Abdeen R (2012) Polyoxypropylene–montmorillonite nanocomposites for drug-delivery vehicles: preparation and characterization. J Appl Polym Sci 125:E157–E166

[63] Elfiky M, Ghoneim M, El-Desoky H, Hassanein A, Salahuddin N (2023) Electrochemical stripping voltammetrical sensor based on polypyrrole exfoliated polyetheramine–montmorillonite nanocomposite for nanomolar detection of Nifuroxazide. RSC Adv 13:5107–5117 36777946 10.1039/d2ra06160k PMC9909373

[64] Hu X, Wang H, Qi S, Su Z, Wang J, Chen K, Li S, Huang X, Luo S, Xie A (2022) Co/C nanomaterial derived from Co metal–organic framework for oxygen evolution reaction, Ionics, 1–9

[65] Elfiky M, Beltagi AM, Abuzalat O (2021) Selective modified stripping voltammetric sensor based on Ce-1, 4-benzenedicarboxylic metal–organic frameworks porous nanoparticles for picomolar detection of curcumin. J Electroanal Chem 898:115606

[66] Heller-Kallai L, Bergaya F, Theng B, Lagaly G (2006) Handbook of clay science

[67] Li J-R, Sculley J, Zhou H-CJCr (2012) Metal–organic frameworks for separations. Chem Rev 112:869–932 21978134 10.1021/cr200190s

[68] Radinović K, Milikić J, Santos DM, Saccone A, De Negri S, Šljukić B (2020) Electroanalytical sensing of trace amounts of As (III) in water resources by Gold–Rare Earth alloys. J Electroanal Chem 872:114232

[69] Bard AJ, Faulkner LR, White HS (2022) Electrochemical methods: fundamentals and applications. Wiley

[70] Kim M-N, Yang J-K, Park Y-J, Lee I-Y, Min K-C, Jeon C, Lee S-M (2016) Application of a novel electrochemical sensor containing organo-modified sericite for the detection of low-level arsenic. Environ Sci Pollut Res 23:1044–1049 10.1007/s11356-015-5747-1 26552791

[71] Erk N, Kurtay Gl, Bouali W, Sakal ZGlm, Genç AAe, Erbaş Z, Soylak M (2024) Electrochemical detection of Melphalan in biological fluids using a g-C3N4@ ND-COOH@ MoSe2 modified electrode complemented by molecular docking studies with cellular tumor antigen P53. ACS Omega 9:21058–21070 38764632 10.1021/acsomega.4c00558 PMC11097377

[72] Lewis A (2017) Precipitation of heavy metals. Sustainable Heavy Metal Remediation: Volume 1: Principles and Processes. Springer, pp 101–120

[73] Wang LK, Vaccari DA, Li Y, Shammas NK Chemical precipitation, Physicochemical treatment processes, Springer2005, pp. 141–197

