# Supplementary material for: Selective detection of arsenite in alkaline media using a yolk-shell (Ce/Fe) terephthalate/organoclay framework stripping voltammetric sensor
Source: Mikrochim Acta. 2026 Mar 17;193(4):246. doi: 10.1007/s00604-025-07782-x (PMC12996381; doi:10.1007/s00604-025-07782-x)
Supplement: Supplementary file 1 — (DOCX 438 KB) [file 604_2025_7782_MOESM1_ESM.docx]

**Selective detection of arsenite detection of arsenite in alkaline media using a yolk-shell (Ce/Fe) terephthalate/organoclay framework stripping voltammetric sensor**

Mona Elfiky^a^*, Amr M. Beltagi^b^

^a^ Department of Chemistry, Faculty of Science, Tanta University, 31527, Tanta, Egypt

^b^Department of Chemistry, Faculty of Science, Kafrelsheikh University, 33516 Kafrelsheikh, Egypt

**Corresponding author e-mail:* [*Elfiky_mona@science.tanta.eu.eg*](mailto:Elfiky_mona@science.tanta.eu.eg)


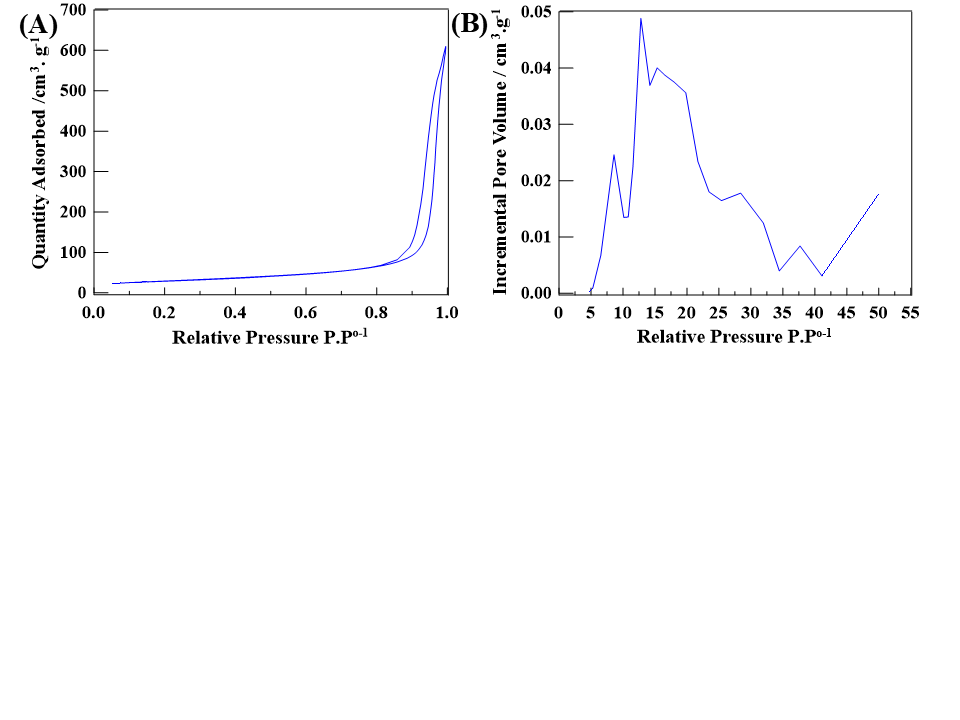


**Fig. S_1._ (A)** N_2_ adsorption-desorption isotherms and **(B)** Dollimore-Heal (DH) method for pore size distribution of Ce/Fe-Tph./MMt_D2000_ framework.


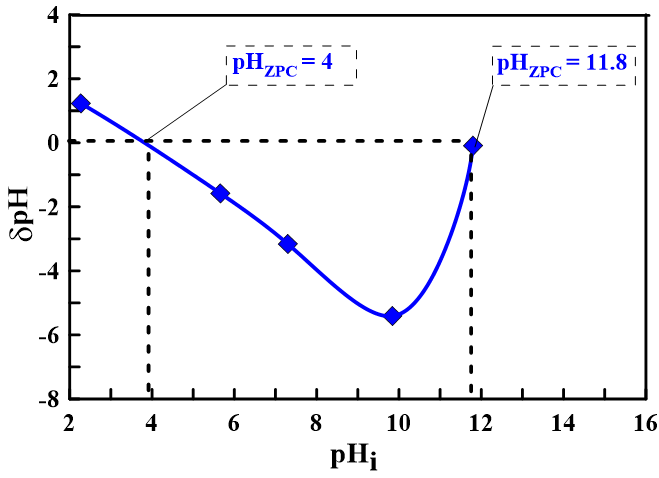


**Fig. S_2._** The plot of ***δpH*** (***pH*_f_**-***pH*_i_**) vs. ***pH*_i_** including the value of ***pH*_ZPC_** of Ce/Fe-Tph./MMt_D2000_ framework.


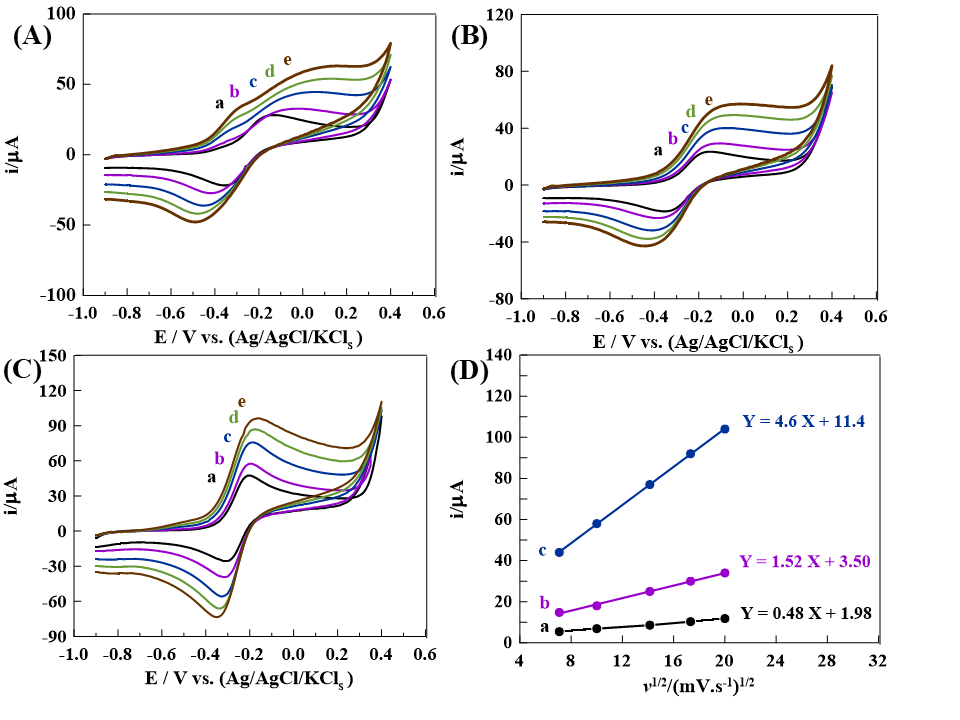


**Fig. S_3._** CV voltammograms of 1.0 mM of K3[Fe(CN6)] in 0.1 M of KClatv ≈ 20- 400 mV·s−1 using (A) BGPS, (B) 1.0 % (Ce/Fe-Tph.), (C) 1.0 % (Ce/Fe-Tph./MMt_D2000_) MGPS (n= 3), and (D) their corresponding *I_p_* vs. ^1/2^ Plot.


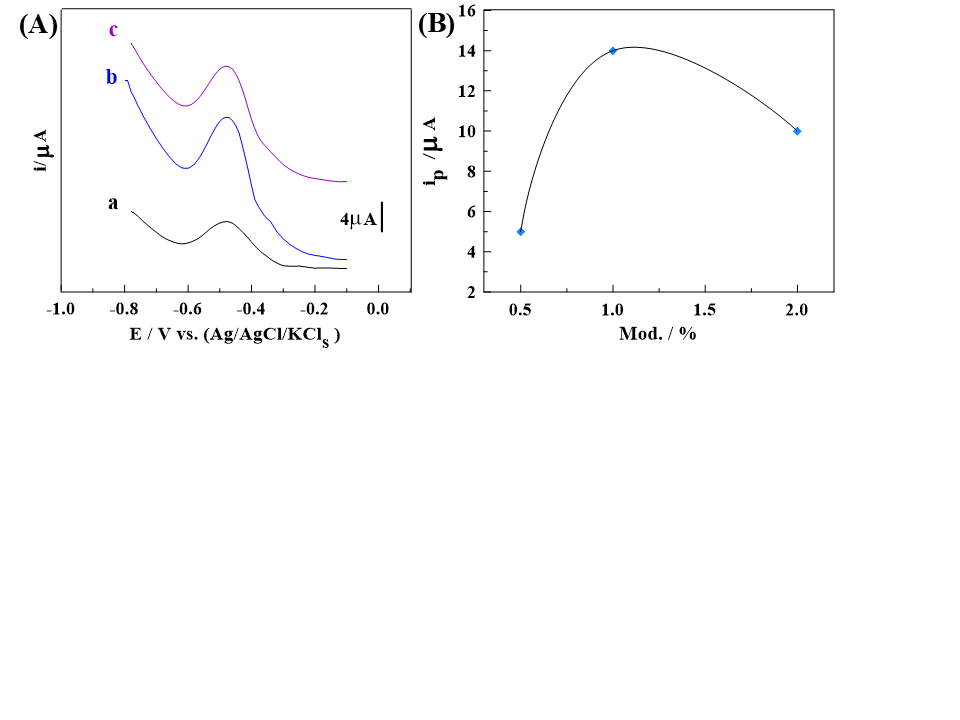


**Fig. S_4._ (A)** SW-AdCSVs of 1.0 nM of arsenite ions in PBS with a pH of 12 recorded at E_acc_= -0.1 V for 35 s using (a) 0.5 % (w/w), (b) 1.0 % (w/w), and (c) 2.0 % (w/w) of [Ce/Fe-Tph./MMt_D2000_] MGPSs, and **(B)** their corresponding (I_p_) plot.


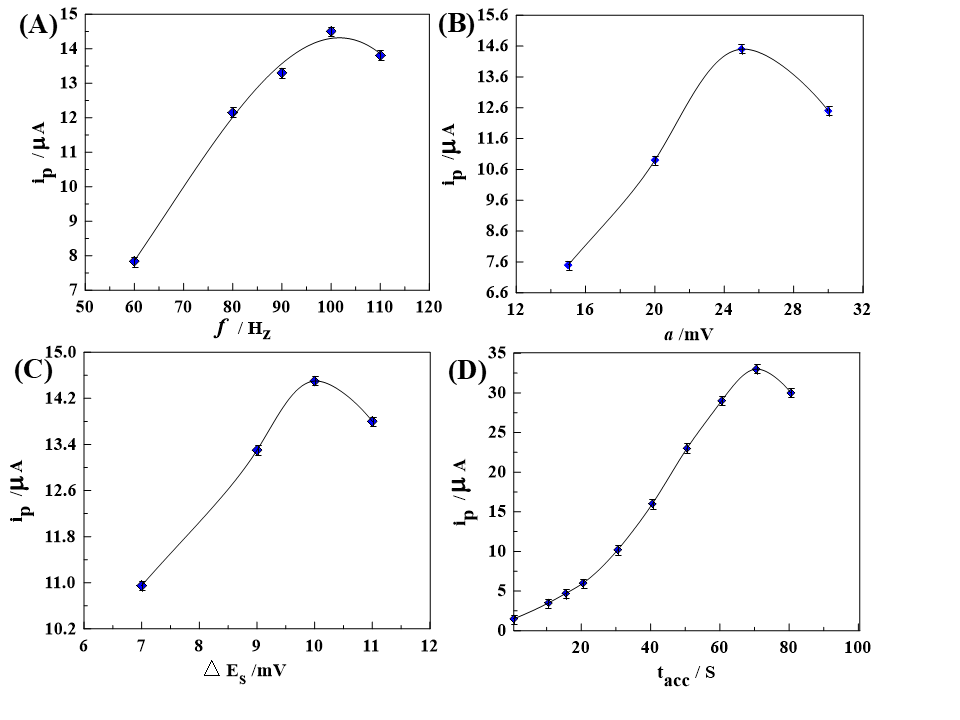


**Fig. S_5._** Influence of **(A)** frequency (*f*), **(B)** pulse amplitude (*a*), and **(C)** scan increment (∆*E_s_*) upon the surface of the 1.0 % (Ce/Fe-Tph./MMt_D2000_) MGPS at*E_acc_* = −0.1 V for 35 s.
